# Using environmental distractors in the diagnosis of ADHD

**DOI:** 10.3389/fnhum.2013.00805

**Published:** 2013-11-22

**Authors:** Hanoch Cassuto, Anat Ben-Simon, Itai Berger

**Affiliations:** ^1^Pediatric Neurology Clinic, Leumit and Clalit HMOJerusalem, Israel; ^2^National Institute for Testing and EvaluationJerusalem, Israel; ^3^Pediatric Division, The Neuro-Cognitive Center, Hadassah-Hebrew University Medical CenterJerusalem, Israel

**Keywords:** ADHD, CPT, visual, auditory, distractibility, diagnosis, validity

## Abstract

This study examined the effect of the incorporation of environmental distractors in computerized continuous performance test (CPT) on the ability of the test in distinguishing ADHD from non-ADHD children. It was hypothesized that children with ADHD would display more distractibility than controls while performing CPT as measured by omission errors in the presence of pure visual, pure auditory, and a combination of visual and auditory distracting stimuli. Participants were 663 children aged 7–12 years, of them 345 diagnosed with ADHD and 318 without ADHD. Results showed that ADHD children demonstrated more omission errors than their healthy peers in all CPT conditions (no distractors, pure visual or auditory distractors and combined distractors). However, ADHD and non-ADHD children differed in their reaction to distracting stimuli; while all types of distracting stimuli increased the rate of omission errors in ADHD children, only combined visual and auditory distractors increased it in non-ADHD children. Given the low ecological validity of many CPT, these findings suggest that incorporating distractors in CPT improves the ability to distinguish ADHD from non-ADHD children.

## Introduction

The diagnosis of Attention Deficit/Hyperactivity Disorder (ADHD) is predominantly based on behavioral symptoms. ADHD is characterized by persistent pattern of inattention and/or hyperactivity-impulsivity, which is maladaptive and inconsistent with a comparable level of developmental age [American Psychiatric Association (APA), 2013]. The DSM criteria classify the disorder into three general presentations– predominantly inattentive, predominantly hyperactive-impulsive, and combined presentation. Children who exhibit the behavioral symptoms of ADHD but demonstrate no functional impairment do not meet the diagnostic criteria (APA, 2013). One of the major difficulties in diagnosis ADHD is that decisions about the inappropriateness of behavior are based on subjective judgments of the observers. Despite efforts of standardization, there are no data to offer a precise estimate of when diagnostic behavior becomes inappropriate (Rader et al., 2009; Berger, 2011). Therefore, the behavioral characteristics remain subjective and maybe interpreted differently by different observers and in different cultures (American Academy of Pediatrics, 2000; Schonwald and Lechner, 2006). Significant variations in the prevalence rates around the world, based on variations in diagnostic methods, support the hypothesis of the role of diagnostic criteria bias (Rousseau et al., 2008).

Since ADHD diagnosis is a complex, multi-factorial task, it requires an integration of data. Typically, the data is assessed by clinical interview and observation, ratings of behavioral scales, and medical-neuro-developmental examination (Wolraich et al., 2011; APA, 2013). In schools and college settings the diagnosis of ADHD may provide additional secondary gains, such as specific academic advantages including additional time to complete assignments and tests, elimination of spelling penalties, advantageous seating in the classroom, testing environments that are free from distractions, etc. Given these benefits, there could be an impetus to feign or simulate the symptoms of ADHD (Sollman et al., 2010). With ADHD information readily accessible on the internet, today's students are likely to be symptom educated prior to evaluation, so ADHD can be readily feigned, particularly when symptoms assessment is based mainly on checklists (Sansone and Sansone, 2011).

Due to these diagnostic complexities and the subjective nature of the assessment instruments, efforts should be made so that the diagnosis of ADHD will be carefully undertaken through the integration of a number of sources of information and sophisticated testing. This attitude might explain the growing use of laboratory-based tools, such as the continuous performance tests (CPT), as complementary strategies in the assessment process.

The visual CPT, which was originally developed as a measure of vigilance and detection of deficits in sustained attention (Rosvold et al., 1956; Rutschmann et al., 1977; Cornblatt et al., 1988), has been widely used and is reported to be the most popular clinic-based measure of sustained attention and vigilance (Edwards et al., 2007). Despite the popularity of the CPT, many studies have questioned its reliability and validity for several reasons (McGee et al., 2000; Edwards et al., 2007; Skounti et al., 2007; Adams et al., 2009). Most CPT are based on a simple visual task that primarily measures the ability of subjects to focus attention and to remain vigilant over time (Shalev et al., 2011).

Typical visual CPT task requires the participant to sustain attention over a continuous stream of stimuli (single letters, shapes, or digits which are presented serially) and to respond to a pre-specified target (Kelip et al., 1997; Shalev et al., 2011). Traditionally, inattention is assessed in CPT by the number of omission errors, indicating the number of times the target was presented, but the participant did not respond, or by its “inverse” measure calculating relative accuracy (the number of correct hits out of the total targets presented). Additional tested measure is the frequency of commission errors, indicating the number of times the participant responded to a non-target stimulus, which is an indicator of impulsivity. Most CPT paradigms assume that ADHD patients become more inattentive as the task progresses, therefore, increasing number of omission and commission errors over time indicate a difficulty to sustain attention over time, namely, to continue process the information effectively (Greenberg and Waldman, 1993). Contextual factors, such as distracting stimuli in the environment, can contribute to increased inattention (Adams et al., 2011). Therefore, sustained attention can be broadly characterized as the ability to concentrate on a specific stimulus over a period of time while excluding distracting stimuli (Shalev et al., 2011). When attending to a target stimulus in the environment, individuals must select the relevant information on which to focus (i.e., attend to the target) while simultaneously ignoring irrelevant information (Godijn and Theeuwes, 2003). Distracting stimuli might, therefore, have an effect on sustained attention by increasing the rate of omission errors in CPT. Therefore, we would expect an ADHD group of children to perform significantly different than non-ADHD peers in a CPT when measuring omission errors.

A major criticisms frequently voiced against the CPT refers to its low ecological validity, that is, the CPT ability to simulate the difficulties of ADHD patients in everyday life (Barkley, 1991; Rapport et al., 2000; Pelham et al., 2011). Being administrated in laboratory conditions (Gutiérrez-Maldonado et al., 2009), most CPT are usually free of distracting stimuli (apart from the non-target stimuli), which are thought to impair the cognitive performance of ADHD children (APA, 1994, 2000). This limitation may explain the loose association between CPT performance and behavioral measures of inattention and hyperactivity, such as those reported by parents and teachers in symptoms rating scales (DuPaul et al., 1992; McGee et al., 2000; Weis and Totten, 2004).

Some efforts have been made to assess distractibility in CPT. Presenting non-target stimuli is one option which is considered very subtle and based mainly on visual performance. In some cases, the CPT confidence index (reflecting the degree to which participants' responses match those of people diagnosed with ADHD) served as a measure of distractibility (Martin et al., 2009). Distractibility was based on the consistency of the response pattern and the degree to which this pattern was typical to ADHD population. However, this measure does not exclusively indicate distractibility but rather could characterize other attentional problems.

Several CPT include specific distractibility tasks. One of the widely used is the FDA approved Gordon Diagnostic System (Gordon and Mettelman, 1987). In the GDS CPT Vigilance task, a series of numbers are shown serially on a front display. The participant is asked to respond as quickly as possible when the number “1” is followed by the number “9.” There are a total of 30 target sequences out of a total of 360 trials. Trials are divided into three blocks consisting of 120 stimuli and 10 target sequences each. The GDS CPT records the number of correct presses, omission and commission errors for both the total test as well as each of the three blocks of trials. In this task, distractors appear as numbers which are presented at a rate of one per second and are exposed for 200 ms each. The test takes approximately 6 min to complete (Kurtz et al., 2001). Although the GDS consistently discriminated ADHD children from control groups, there are mixed evidences regarding its ability to discriminate children with ADHD from various disordered controls and its associations with other measures of ADHD. The effect of distractors on its abilities is not clear (Christensen and Joschko, 2001).

Recently, Uno et al. (2006) developed a noise-generated CPT, which included neutral, geometric target/non-target stimuli and auditory/visual distractors (tone or irrelevant letter). This study found that while auditory noise strongly reduced impulsive and inattentive behaviors in ADHD relatively to non-ADHD children, visual distractors decreased the number of omission errors in ADHD children but increased it in non-ADHD children. However, the ecological validity of these trials is questionable due to the use of neutral stimuli. It has been suggested that ADHD children are more distracted when confronting with appealing, reinforcing or emotionally-loaded stimuli than with neutral ones (Blakeman, 2000; López-Martín et al., 2013).

Following the recommendation of Barkley (1991) and others (Rapport et al., 2000; Pelham et al., 2011) to improve the ecological validity of the CPT by evaluating the child's behaviors in more natural settings, virtual reality technologies incorporated typical stimuli of the learning environment (e.g., pencils dropping, chairs moving, airplane flying) into the CPT (Rizzo et al., 2006; Parsons et al., 2007; Adams et al., 2009). These methods consistently identified distractibility in ADHD children, probably due to better simulation of everyday life. However, these CPT tasks require sophisticated technologies that rarely exist in clinical and diagnostic settings.

Up to date, distractibility symptoms are clinically and empirically assessed by a large variety of cognitive tasks, such as Digit Span Distractibility Test (Oltmanns and Neale, 1975), Flanker task (Botvinick et al., 1999), Filter Task (Ophir et al., 2009), or Delayed Oculomotor Response task (Adams et al., 2011). The majority of these tasks involve a competition of responses, so that the child has to inhibit his response to the irrelevant stimuli. These tasks were criticized for their low ecological validity (Blakeman, 2000; van Mourik et al., 2007) because in everyday life, the child has to ignore a stimuli that is external to the task and not conflicting with task demands (e.g., a child is doing schoolwork while someone talks in the next room). Importantly, it is possible that when the distractors compete with the central task, reduced performance in ADHD could be a result of a greater difficulty in inhibiting conflicting stimuli that are incorporated in a task, rather than higher sensitivity to irrelevant stimuli. Separate reviews found that auditory-sustained attention on a CPT (Gentilini et al., 1989; Parasuraman et al., 1991) and verbal-sustained attention with the Paced Auditory Serial Attention Task (Gronwall, 1989) were impaired after mild traumatic brain injury (mTBI).

Taken together, the described findings may suggest that including meaningful and relevant distracting stimuli in CPT may improve its ecological validity.

The objective of this study was to examine the added value of incorporating everyday life visual and auditory distractors into a visual CPT and the effect of the distractors on the ability of the CPT to discriminate ADHD from non-ADHD children. Using the rate of omission errors as an index of sustained attention, this study examined whether ADHD children are more distracted than non-ADHD children. We also examined if and which type of distractors improves the ability of the test to distinguish ADHD from non-ADHD children. In order to examine these questions, this study used a visual CPT which includes environmental distracting stimuli (MOXO-CPT; Berger and Goldzweig, 2010). We hypothesized that several factors may make the MOXO-CPT preferable in terms of ecological validity. First, it includes environmental auditory and visual stimuli that are typical of childrens' everyday life. In contrast to the majority of cognitive tasks, distracting stimuli in the MOXO-CPT are external to the task (i.e., not conflicting with task demands). This method allows measuring the sensitivity of ADHD children to irrelevant stimuli in the classroom (e.g., someone talking in the next room) rather than background stimuli (e.g., music) or distractors that are part of the cognitive task (van Mourik et al., 2007). Finally, this CPT is a standard computerized task which is highly available in clinical practices.

## Methods

### Participants

Participants were 663 children aged 7–12 years, 405 of which were boys and 258 were girls. The clinical group was composed of 345 children previously diagnosed with ADHD (Mean age, 9.39, *SD* = 1.57) and the control group was composed of 318 children without ADHD (Mean age = 9.48, *SD* = 1.58).

Participants in the ADHD group were recruited from children who were referred to out-patient pediatric clinics of a Neuro-Cognitive Center, based in a tertiary care university hospital. The referrals to the center were made by pediatricians, general practitioners, teachers, psychologists, or parents. The following were the inclusion criteria for participants in the ADHD group:

Each child met the criteria for ADHD according to DSM-IV-TR criteria (APA, 2000), as assessed by a certified pediatric neurologist. The diagnostic procedure included an interview with the child and parents, medical/neurological examination and filing of ADHD diagnostic questionnaires (DuPaul et al., 1998).

Participants in the control group were randomly recruited from regular primary school classes. The inclusion criteria for participants in the control group were: (1) score below the clinical cutoff point for ADHD symptoms on ADHD/DSM-IV Scales (DuPaul et al., 1998; APA, 2000) and (2) absence of academic or behavioral problems based on parents and teachers reports.

The exclusion criteria for all participants were: intellectual disability, other chronic condition, chronic use of medications, and primary psychiatric diagnosis (e.g., depression, anxiety, and psychosis). All participants (both groups) studied in regular classes in regular schools.

All participants agreed to participate in the study and their parents provided a written informed consent to the study, approved by the Helsinki committee (IRB) of Hadassah-Hebrew University Medical Center Jerusalem, Israel.

### Tools

#### The MOXO continuos performance test

**Figure 1:**
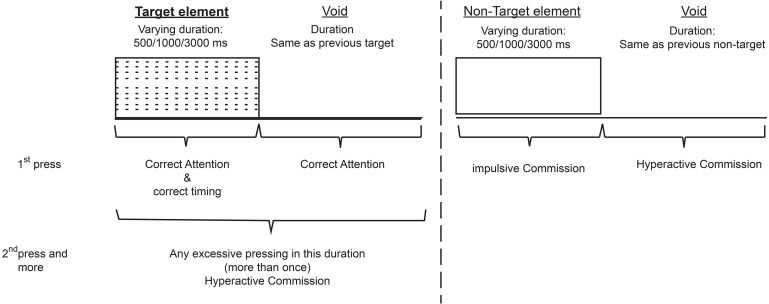
**Definition of the time line- Target and non-target stimuli were presented for 500, 1000, or 3000 ms**. Each stimulus was followed by avoid period of the same duration. The stimulus remained on the screen for the full duration regardless the response. Distracting stimuli were not synchronized with target/non-targel's onset and could be generated during target/non-target stimulus or during the void period.

The current study employed the MOXO-CPT version (Berger and Goldzweig, 2010). The MOXO-CPT (Neuro Tech Solutions Ltd.) is a standardized computerized test designed to diagnose ADHD related symptoms. As in other CPT, the MOXO-CPT task requires a participant to sustain attention over a continuous stream of stimuli and to respond to a pre-specified target, but it also includes visual and auditory stimuli serving as measurable distractors. The test consists of eight stages (levels). Each level consists of 53 trials and lasts 114.15 s. The total duration of the test is 15.2 min. In each trial a stimulus (target or non-target) is presented in the middle of the computer screen for a duration of 0.5, 1, or 3 s and is followed by a “void” of the same duration (see Figure 1). Fifty-three stimuli are presented in each level, of which 33 are target stimuli and 20 are non-target. Each stimulus remains on the screen for the full duration of the designated presentation time, regardless whether a response was given or not. This practice allows the measuring of the timing of the response as well as its accuracy.

The screen size is 125 high and 166 wide. The child is located 60 cm from the screen and is instructed to respond to target stimulus as quickly as possible by pressing the space bar once, and only once. The child is also instructed not to respond to any other stimuli but the target, and not to press any other key but the space bar.

**Figure 2:**
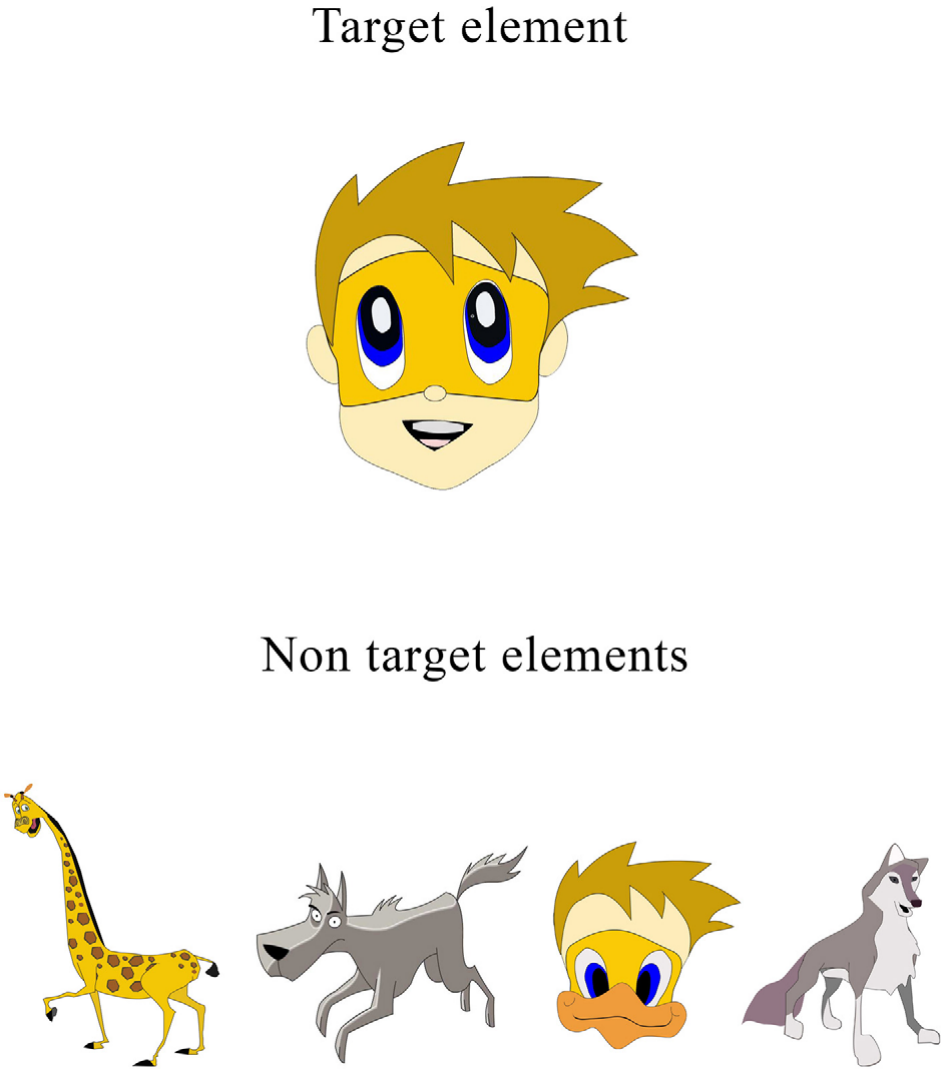
**MOXO-CPT target and non-target stimuli**.

***Test Stimuli.*** Target and non-target stimuli—Both target and non-target stimuli are cartoon pictures free of letters or numbers (see Figure 2). The absence of letters and numbers in the stimuli is important given the fact that ADHD children tend to have learning difficulties (e.g., dyslexia, dyscalculia) that may be confound with CPT performance (Seidman et al., 2001). Target stimulus is always a cartoon image of a child's face. Non-target stimuli include five different images of animals (Figure 1). Both target and non-target stimuli are 41*41 mm large and are always presented in the center of the screen.

**Figure 3:**
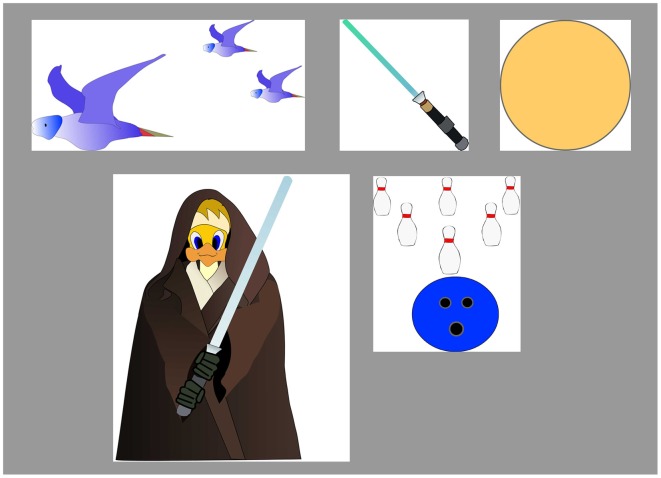
**MOXO-CPT visual distractors**.

Distracting stimuli—To simulate everyday environment, the MOXO-CPT included visual and auditory distracting stimuli which are not part of the non-target stimuli. The distracting stimuli are of various degrees of similarity to the target stimulus. Distractors were short animated video clips containing visual and auditory features which can appear separately or together. Overall, six different distractors were included, each of them could appear as pure visual (e.g., three birds moving their wings), pure auditory (e.g., birds singing), or as a combination of them (birds moving their wings and singing simultaneously). Each distractor was presented for a different duration ranging from 3.5 to 14.8 s, with a fixed interval of 0.5 s between two distractors. Visual distractors (Figure 3) included six different stimuli: a gong (presented for 6.8 s), a bowling ball (3.5 s), birds (9.25 s), warrior (Jedi) with a saber (14.8 s), a saber (6.8 s), and a flying airplane (8.6 s).

Visual distractors appeared at one of four spatial locations on the sides of the screen: down, up, left, or right. Visual distractors that appeared on the left/right axis were 200–400 pixels high and 100–200 wide. Visual distractors that appeared on the up/down axis were 100–200 pixels high and 100–600 wide. The distance between visual distractors and target/non-target stimuli is always 21 mm.

Auditory distractors included the six corresponding sounds of each visual distractor (e.g., a gong sound, sound of a bowling ball, birds singing etc.). The sound is delivered through loudspeakers located on both sides of the screen (about 60 cm distance from the child's ears). The sound intensity was about 70% of the maximal intensity of the loudspeakers. Distractors' onset was not synchronized with target/non-target's onset and could be generated during target/non-target stimulus or during the void period. All distracted were elements which characterize a typical child environment. This feature is unique to the MOXO-CPT in comparison to other CPT.

***Test levels.*** The test comprised of eight levels, with 53 trials in each level. The stimuli and their presentation time are identical across all levels; however, the levels differ in the visual and auditory distractors present in the trials. Different levels of the MOXO-CPT were characterized by a different set of distractors: levels 1 and 8 did not include any distractors but only target and non-target stimuli, levels 2 and 3 contained pure visual stimuli, levels 4 and 5 contained pure auditory stimuli, and levels 6 and 7 contained a combination of visual and auditory stimuli. The sequence of distractors and their exact position on the display were constant for each level. The load of the distracting stimuli increased in the odd number levels: during the 2nd, 4th, and 6th levels only one distractor was presented at a time. During the 3rd, 5th, and 7th levels two distractors were presented simultaneously.

***Performance indices.*** The MOXO-CPT includes four performance indices, the current study focuses on the rate of omission errors as an index of sustained attention:

**Attention**: the number of correct responses (pressing the key in response to a target stimulus), given either during the stimulus presentation on the screen or during the following void period. The difference between the total number of the target stimuli and the number of correct responses produced the number of omission errors.
**Timing**: the number of correct given only while the target stimulus was still presented on the screen.
**Impulsivity**: the number of commission errors (responses to a non-target stimulus).
**Hyperactivity**: the number of all types of commission responses that are not coded as impulsive responses (e.g., multiple responses- pressing the keyboard's space bar more than once to target or non-target, random key pressing—pressing other keyboard button than the space bar). For more detailed description of performance indices see Appendix.

In this research we focused mainly on the index of omission errors. This index measures the number of times the child did not respond to target stimuli either during the stimulus presentation or during the void time. Hence, it can be regarded as a pure measure of difficulty in sustained attention which is not dependent on response speed.

### Procedure

The MOXO-CPT was administered by a technician who made sure that the child understood the instructions. The technician was present throughout the entire session. The examination room was clear of other distractors. All children (including the ADHD group) were drug naïve while performing the test.

### Data analyses

All analyses were carried out using the SAS software for Windows version 9.2. First, Chi-square analysis and *t*-test for unpaired samples were used to examine group differences in background variables. Second, effects of background variables, ADHD, and test level on omission errors were examined through a Linear Repeated Measures model with Tukey's correction for multiple comparisons. Omission errors were the dependent variable, whereas age, gender, group, level were the independent variables.

#### In addition, level ^*^ group interaction was calculated

Between and within group effects were measured in every CPT condition (no distractors, visual distractors, auditory distractors, and a combination of visual and auditory distractors). For this purpose, every two identical levels were combined: levels 1 and 8 (no distractors), levels 2 and 3 (visual distractors) levels 4 and 5 (auditory distractors), and levels 6 and 7 (combination of visual and auditory distractors).

## Results

### Background variables

The two groups did not differ in age [*t*_(661)_ = −0.81, *p* = 0.42] but the percentage of boys in the ADHD group (68%, *N* = 235) was significantly higher than in the control group (54%, *N* = 172) [χ^2^(1, *N* = 663) = 13.15, *p* < 0.001]. However, when the effect of gender on omission errors was examined using a Linear Repeated Measures model, gender did not have a significant effect [*F*_(1, 659)_ = 1.05, *p* = 0.31].

### Effects of distractors on omission errors in ADHD and non-ADHD children

In order to study the added value of the incorporation of distractors in the CPT for a better differentiation between AHDH and controls a linear repeated measures model with Tukey's correction for multiple comparisons was conducted.

This model included (a) between groups analysis of the differences in the rate of omission errors between ADHD and non-ADHD children, and (b) within-group analysis of the differences in omission errors between no distractors conditions and the three conditions which contained distractors (visual, auditory, and a combination of them).

First, analyses showed that while gender was not associated with CPT performance, age had a significant effect on it [*F*_(1, 659)_ = 97.59, *p* < 0.001].

When controlling for age and gender, group affiliation had a significant effect on the rate of omission errors [*F*_(1, 659)_ = 92.59, *p* < 0.001]. As can be seen in Table 1, ADHD children demonstrated higher rate of errors than non-ADHD children in all CPT conditions (no distractors, visual distractors, auditory distractors, and a combination of visual and auditory distractors). Most importantly, group ^*^ level interaction revealed that the differences between the two groups varied as a function of the task demands [*F*_(3, 659)_ = 15.55, *p* < 0.001]. Within-groups analysis indicated that for the ADHD group, omission errors were significantly higher in all distractors conditions compared to no-distractors. However, in the control group, only combined distractors resulted in an increase in omission errors (Table 2).

**Table 1:** **Differences in Omission errors between ADHD and non-ADHD Children**.

**Level's number**	**Distractors type**	**ADHD (***N*** = **345**)**		**Control (***N*** = **318**)**		**Difference *t* (659)**
		**Omission errors**		**Omission errors**		
		***M***	***SD***	***M***	***SD***	
1	Base line	1.80	2.57	0.80	1.30	6.18, *p* < 0.001
2	Visual^a^	3.21	3.38	1.19	1.32	10.53, *p* < 0.001
3	Visual^b^	2.73	3.09	1.18	1.42	8.46, *p* < 0.001
4	Auditory^a^	2.50	3.21	0.95	1.25	8.26, *p* < 0.001
5	Auditory^b^	2.74	3.86	0.97	1.39	7.84, *p* < 0.001
6	Combined^a^	3.52	3.90	1.58	1.64	8.50, *p* < 0.001
7	Combined^b^	3.45	4.17	1.75	2.17	6.57, *p* < 0.001
8	No distractors	2.26	3.19	1.21	1.95	5.01, *p* < 0.001

a Low distractibility;

b High distractibility; M, mean; SD, standard deviation.

**Table 2:** **Level differences in Omission errors within each study group**.

**Level's number**	**Distractors type**	**ADHD (***N*** = **345**)**			**Control (***N*** = **318**)**		
		**Omission errors**		**Difference from baseline level *t*_(659)_**	**Omission errors**		**Difference from baseline level *t*_(659)_**
		***M***	***SD***		***M***	***SD***	
1	Base line	1.80	2.57		0.80	1.30	
2	Visual^a^	3.21	3.38	−12.51, *p* < 0.001	1.19	1.32	−3.27, *p* = 0.08
3	Visual^b^	2.73	3.09	−8.46, *p* < 0.001	1.18	1.42	−3.31, *p* = 0.07
4	Auditory^a^	2.50	3.21	−6.04, *p* < 0.001	0.95	1.25	−1.23, *p* = 0.99
5	Auditory^b^	2.74	3.86	−6.63, *p* < 0.001	0.97	1.39	−1.11, *p* = 0.99
6	Combined^a^	3.52	3.90	−12.09, *p* < 0.001	1.58	1.64	−5.20, *p* < 0.001
7	Combined^b^	3.45	4.17	−10.06, *p* < 0.001	1.75	2.17	−5.53, *p* < 0.001
8	No distractors	2.26	3.19	−3.45, *p* = 0.05	1.21	1.95	−2.94, *p* = 0.20

a Low distractibility;

b High distractibility; M, mean; SD, standard deviation.

## Discussion

This study investigated the effects of environmental distractors on sustained attention of ADHD and non-ADHD children. Results showed that while ADHD children were negatively impacted by all types of distractors (visual, auditory, and a combination of them) non-ADHD children were affected only by the combination of visual and auditory stimuli. This finding confirms the sensitivity of ADHD children to environmental distracting stimuli and is consistent with other studies demonstrating higher distractibility of ADHD children in a variety of cognitive tasks (Adams et al., 2011; Pelham et al., 2011).

It is known that a variety of visual and auditory stimuli exists in the everyday environment of ADHD children and that problematic behavior first appear in the presence of such stimuli. Thus, our results support the idea that ADHD is indeed marked by high distractibility and that children with ADHD have difficulties to sustain attention in the presence of irrelevant environmental stimuli. These findings are in line with other studies that demonstrated higher distractibility of ADHD children during CPT and non-CPT tasks (Gumenyuk et al., 2005; Parsons et al., 2007; Adams et al., 2011; Pelham et al., 2011). Parsons et al. (2007), who used a virtual reality technology to simulate everyday distractibility in ADHD, have shown that during distracting conditions, ADHD children were more hyperactive and produced more omission errors on the Conners' CPT-II as compared to non-ADHD children. Likewise, Gumenyuk et al. (2005) shown that when a novel sound appeared during a visual discrimination task, ADHD children showed higher rate of omission responses and different patterns of event-related potentials (ERP) (smaller amplitude over the fronto-central left-hemisphere during the early phase of P3a and a larger amplitude during its late phase compared to controls). These findings were attributed to the deficient control of involuntary attention in ADHD children that may underlie their abnormal distractibility.

On the other hand, our findings are inconsistent with other studies which indicated that auditory and visual distractors did not impair cognitive performance of ADHD children or even improved it (Abikoff et al., 1996; Uno et al., 2006; Söderlund et al., 2007; van Mourik et al., 2007; Pelham et al., 2011). Uno et al. (2006) who specifically tested the effect of auditory noise on CPT performance, found that ADHD children produced fewer omission errors in the presence of auditory noise than in the no-noise condition. Similarly, van Mourik et al. (2007) found that the occurrence of an irrelevant, novel sound prior to a visual stimulus decreased the rate of omission errors in ADHD children relatively to no-sound conditions. The positive effect of distracting auditory stimuli on the cognitive performance of ADHD patients is usually attributed to the increased arousal provoked by a novel signal (Uno et al., 2006; van Mourik et al., 2007). It is possible that distractors in the MOXO-CPT failed to improve attention in ADHD children because of the little information they conveyed for the participant. It has been suggested (Parmentier et al., 2010) that the degree to which a novel, unexpected auditory sound may optimize performance depends on the amount of information it conveys. When a novel sound predicts another relevant stimulus, the system can take advantage of the auditory distractors to improve its functioning. In contrast to other CPT tasks (e.g., Uno et al., 2006; van Mourik et al., 2007), distractors in the MOXO-CPT did not precede the target or were generated simultaneously with it, but rather were unsynchronized with it. This fact may lower the extent to which the sound included information necessary to optimize performance and may explain why auditory distractors did not improve sustained attention in our study.

The diversity of our results from the studies mentioned above could also result from the type of distractors used. While some studies have used neutral stimuli (neutral tone/letter) as distractors (Gordon and Mettelman, 1987; Uno et al., 2006), the MOXO-CPT used more ecologically valid stimuli that are typically found in the child's environment. Since ADHD children have more difficulties in filtering meaningful distractors (Blakeman, 2000) they may fail to inhibit response to relevant or appealing stimuli. Another factor that may contribute to the high distractibility of ADHD children in this study is the method of distractors presentation. In several studies, auditory distractors served as a background noise while children performed another cognitive task (Abikoff et al., 1996; Pelham et al., 2011). In contrast, distractors in the MOXO-CPT vary in their type, in their length of presentation and in their location on the screen. This mode of presentation did not allow adjustment or de-sensitization to the distractors, therefore, kept them salient. Finally, methodological differences in distractors presentations may underlie the contrasting findings. While Gumenyuk et al. (2005) used headphones to present auditory distractors; van Mourik et al. (2007) used a speaker. It is possible that auditory stimuli served as more potent distractors to ADHD children when presented by headphones than by a speaker because they drew the patient's attention spatially away from the visual targets. However, this argument is not enough to explain why auditory stimuli in the MOXO-CPT distracted ADHD children despite the use of a loudspeaker.

It can be argued that because levels in the MOXO-CPT are presented in a constant manner (namely level 1 to level 8), distractors' effect may be confounded with time effects. However, our findings suggest that it might not be the case. In the current study children in the control group did not perform more omission errors at the last level of the test (level 8) than at the first one (level 1). ADHD children performed marginally more omission errors in the last level (level 8), but it is a rather weak/marginal effect which does not seem a cause for concern. Moreover, in both groups, there was no linear increase in omission errors as the test progressed as we would expect if time was negatively associated with sustained attention.

The finding that both ADHD and control groups did not perform significantly more omission errors at the end of the test than at the beginning of it (i.e., both groups did not demonstrate clear fatigue effects) is in contrast to other studies indicating degraded performance in ADHD patients as the task progresses (McGee et al., 2004; Erdodi et al., 2010; Erdodi and Lajiness-O'Neill, 2013). According to Huang-Pollock et al. (2012), degraded performance over time in ADHD patients is more prominent in tasks that use sensory stimuli (i.e., discriminations of physical characteristics that differ in degree) than in tasks that use cognitive stimuli (i.e., stimuli that differ in kind, such as alphanumeric symbols) because of the lower level of effort in the latter type. *The fact that the MOXO-CPT, like most CPT tasks, relied on cognitive stimuli may explain why we failed to identify time effects on performance*. Nevertheless, time effect on CPT performance should be further addressed in future studies.

Several limitation of this study should be considered. First, participation in the study was based on a voluntary agreement of children and their parents. This self-selected sampling strategy tends to be biased toward favoring more cooperative and motivated individuals. Therefore, it is not possible to determine whether this sample also represents other children that were not recruited and whether cooperation is confounded with ADHD variables. This limitation is typical to most clinic-based ADHD studies around the world (Lee and Ousley, 2006; Gau et al., 2010). In addition, the clinics from which ADHD children were recruited were based in tertiary care hospital. Finally, the exclusion of ADHD children with severe comorbidities may limit the generalization of our results.

In light of the criticism voiced against the low ecological validity of many CPT (Rapport et al., 2000; Pelham et al., 2011), the current study provides evidence that adding environmental distractors to CPT impaired the ability of ADHD children to sustain attention and strongly increased their omission errors as compared to non–ADHD children. For non-ADHD children only a combination of visual and auditory stimuli created enough cognitive load to impair attention.

Future research should address the diagnostic utility of the test in larger spectrum of age, in samples with comorbid features, and in different sub-types of ADHD. In addition, future research should investigate the effects of medical treatment on the distractibility of ADHD children.

## Author contributions

All authors contributed extensively to the work presented in this paper.

### Conflict of interest statement

Itai Berger serves in the scientific advisory board to Neuro-Tech Solutions Ltd. Anat Ben-Simon and Hanoch Cassuto declared no potential conflicts of interest with respect to this article.

## Appendix

### Description of performance indices

#### Attention

This parameter included the number of correct responses (pressing the key in response to a target stimulus), which were performed either during the stimulus presentation on the screen or during the void period that followed. Thus, it was possible to evaluate whether the participant responded correctly to the target (was attentive to the target) independently of how fast he was. Knowing how many responses are expected, it was also possible to calculate the number of times the target was presented, but the patient did not respond to it (omission errors).

#### Timing

This parameter included the number of correct responses (pressing the key in response to a target stimulus) which were performed only while the target stimulus was still presented on the screen. This parameter did not include responses that were performed during the void period (after the stimulus has disappeared). According to the National institute of mental health (2012), inattention problems in ADHD may be expressed in “difficulties in processing information as quickly and accurately as others.” Traditionally, difficulties in timing at a CPT are evaluated by mean response time for correct responses to the target (which is interpreted as a measure of information processing and motor response speed) and by the standard deviation of response time for correct responses to the target (which is interpreted as a measure of variability or consistency) (Greenberg and Waldman, 1993). In these paradigms the stimulus is presented for short and fixed periods of time and the response occurs after the stimulus has disappeared. Given the short, fixed presentation, accurate but slow participants may be mistakenly diagnosed as inattentive. While a group of patients would respond correctly if allowed more time, inattentive patients would not respond at all because they were not alert to the target. Therefore, the measurement of response time *per-se*, addresses only the ability to respond quickly, but not the ability to respond accurately. By implanting a void period after each stimulus and using variable presentation durations of the elements, the MOXO-CPT could distinguish accurate responses performed in “good timing” (quick and correct responses to the target performed during stimulus presentation) from accurate but slow responses (correct responses to the target performed after the stimulus presentation; during the void period). These two aspects of timing correspond to the two different problems of ADHD described by the National institute of mental health (2012); responding quickly and responding accurately.

#### Impulsivity

This parameter included the number of commission errors (responses to a non-target stimulus), performed as responses to the non-target stimuli. Usually, commission errors are coded in any case of inappropriate response to the target (e.g., pressing a random key) (Greenberg and Waldman, 1993). In contrast, the MOXO-CPT's impulsivity parameter considered as impulsive behavior only the pressings on the keyboard's space–bar in response to non-target stimulus. All other non-inhibited responses (e.g., pressing the keyboard more than once) were not coded as impulsive responses (as will describe in the next paragraph).

#### Hyperactivity

This parameter included all types of commission responses that are not coded as impulsive responses. Several examples are: (1) Multiple responses- pressing the keyboard's space bar more than once (in response to target/non-target), which is commonly interpreted as a measure of motor hyper-responsivity (Greenberg and Waldman, 1993). The MOXO-CPT considered as multiple responses only the second press and above (the first response would be considered as correct response with good timing, as correct response with poor timing, or as impulsive response, depends on the type of element appearing on the screen). (2) Random key pressing—pressing any keyboard button other than the space bar. By separating commission errors due to impulsive behavior from commission errors due to motor hyper-responsivity, it was possible to identify the multiple sources of response inhibition problems. Thus, the MOXO-CPT was able to differentiate impulsive responses from hyperactive responses.
